# Technical Quality of Root Canal Filling in Preclinical Training at Strasbourg University Using Two Teaching Protocols

**DOI:** 10.1055/s-0039-1698848

**Published:** 2019-12-31

**Authors:** Naji Kharouf, Joseph Hemmerlé, Youssef Haikel, Davide Mancino

**Affiliations:** 1 Department of Endodontic and Conservative Dentistry, Faculty of Dental Medicine, Strasbourg University, Strasbourg, France; 2 Inserm UMR_S 1121, Biomaterials and Bioengineering, Strasbourg, France

**Keywords:** canal filling, mechanical preflaring, pre-access analysis, step-down technique, cold hydraulic condensation

## Abstract

**Objectives** The aim of this study was to compare two teaching protocols according to the technical quality of root canal therapy (RCT) and the procedural errors occurred in preclinical training.

**Materials and Methods** Two different groups of students were concerned. The first one (G1) performed a crown-down technique to shape the root canal systems and cold lateral condensation technique to fill them. The second one (G2) performed a step-down technique without initial manual scouting to shape the root canal systems, and cold hydraulic condensation technique, to fill them. G2 used clinical operative microscope to check the access cavity preparation.

**Statistical Analysis** The quality of RCTs and procedural errors were recorded and analyzed using chi-squared test and *t* -test.

**Results** Four hundred sixty-eight root canals from 152 maxillary molars were treated by the G1 students: 46.6% canals were judged as acceptable. Four hundred sixty-nine root canals from 152 mandibular molars were treated by G1: 58.8% canals were judged as acceptable. Five hundred fifteen root canals from 156 maxillary molars were treated by G2 students: 84.1% canals were judged as acceptable. Four hundred ninety-three root canals from 156 mandibular molars were treated by G2: 90.9% canals were judged as acceptable. Among the errors, the incidence of “ledges” and “fractured instruments” was statistically significant in G1 compared with G2, both on maxillary and on mandibular molars.

**Conclusions** The molar RCTs performed by G2, who got benefit from the new teaching protocol, resulted in a better quality of root filling and in fewer procedural errors compared with the molar RCTs performed by G1.

## Introduction

Many epidemiological studies report a strong association between apical periodontitis and inadequate technical quality of root filling. Furthermore, they show unsatisfactory quality standards of root canal therapy (RCT) performed in general practice and in dental teaching hospitals. 1 2 3 4 5 6

In France, like in a lot of others European countries, endodontics is not a specialty, and the majority of endodontic procedures are performed by general practitioners (GP) and particularly by young graduated dentists. They rarely respect the basic principles to correctly perform an endodontic therapy, probably even as a result of a low standard of undergraduate training. 7 8

It is crucial that undergraduate dental students may get benefit from the teaching and clinical supervision of endodontic specialists. 9 New technologies should be used to train students and allow them to be able to perform regularly and correctly an endodontic therapy, 10 even in multirooted teeth, as the European Society of Endodontology (ESE) undergraduate curriculum guidelines recommend. You have to consider that the majority of current undergraduate students will be the GPs of tomorrow. 8 Even though the introduction of new technologies, like surgical microscopes and apex locators, could be a serious problem in many dental universities because of the expensive materials.

This study was conducted using exclusively the radiographic analysis. Its aim was to compare two teaching protocols according to the technical quality of root canal filling and the procedural errors occurred in preclinical training. We wanted to point out if educational changes could result in higher-quality RCT in preclinical training.

## Materials and Methods

This study was revised and approved by the Ethics Committee, of Medical, Odontology School, and Strasbourg University Hospital (protocol no. 2018–89).

We investigated the quality of RCTs, on extracted molars, performed by 3rd year undergraduate dental students in preclinical training during their final exams, using two different protocols at Strasbourg University (SU).

Before the exams, the students of both groups got benefit from the same number of lectures and training courses on extracted teeth using the specific method performed in their group.

Each group had 14 training courses, 7 for mandibular, and 7 for maxillary molars before passing the practical exams. Each training course lasted 4 hours.

For G1, these 4 hours were spent to perform RCTs. Whereas each training course of G2 was divided in two sections: 3 hours were spent to perform RCTs and 1 hour to analyze and discuss some RCTs performed during the same session.

In their preclinical training exams, G1 treated 468 maxillary molar canals and 469 mandibular molar canals using the crown-down technique for the root canal shaping and the cold lateral condensation (CLC) technique for the root canal filling, using master gutta-percha point, accessory cone, and AH plus sealer. This protocol was taught by an experienced GP at SU until 2016 to 2017.

In their preclinical training exams, G2 treated 515 maxillary molar canals and 493 mandibular molar canals using a step-down technique without initial manual scouting for the shaping and the cold hydraulic condensation (CHC) technique for the root canal filling, using matched gutta-percha point and BioRoot (Septodont; Saint-Maur-des-Fossés, France) as bioceramic sealer. This second protocol was taught by an endodontic specialist at SU in 2017 to 2018.

Each student had a set of new SS and NiTi instruments to treat a single extracted molar. All the radiographs were taken using a phosphor plate (Dürr Dental AG; Bietigheim-Bissingen, Germany). The radiographs analyzed for this study were taken by the students for their final preclinical training exams and not intentionally for this study.

The following steps were performed using the crown-down technique: 11 12

1. Analysis phase: It was performed taking one preoperative radiograph.
2. Access opening phase: It was performed using a 016 cylindric diamond bur and an Endo Z Tungsten Carbide Bur.
3. Relocating phase: It was performed systematically using SX or Gates Glidden Drills number 3, with an intentional brushing motion, without exceeding 3 mm of depth.
4. Scouting phase: It was performed using K10 until to first resistance.
5. Enlargement phase: It was performed using Proper gold S1 with a brushing motion and, no deeper than the level of the penetration of the scouting file less 1 mm.
6. Working length (WL) phase: It was performed using K10 until to radiographic WL (RWL).
7. Manual glide path phase: It was performed using K15 until to RWL.
8. Shaping phase: It was performed using ProTaper gold instruments S1, S2, F1, F2 to RWL.
9. Irrigation phase: It was performed using 2.5% NaOCl, syringe and 27-gauge needle.
10. Intraoperative radiograph with master cones at WL.
11. Filling phase: It was performed using cold lateral technique with a master cone coated with a thin coat of AH plus sealer and medium-fine GP accessory points.
12. Postoperative radiograph.

The following steps were performed using the technique step-down technique without initial manual scouting: 13

1. Analysis phase: It was performed taking two preoperative radiograph.
2. Access opening phase: It was performed using a 016 cylindric diamond bur and an Endo Z Tungsten Carbide Bur.
3. Checking phase: It was performed using an operative microscope (Zumax, Jiangsu, China) to check the access cavity preparation.
4. Preflaring phase: Initial rotary preflaring until to two-thirds of estimated root canal length or until to the first impediment, using Proglider (Dentsply Sirona; York, Pennsylvania, United States).
5. Apical scouting phase: apical scouting until RWL + 0.5 mm using a SS K10 file (intraoperative radiograph).
6. Glide path phase: Rotary glide path until RWL, using Proglider.
7. Shaping phase: It was performed using ProTaper gold instruments and if necessary Profile (PF) 25/04 and 25/06; Basically S1, S2, F1 and PF 25/04 or PF 25/06 to RWL to shape mesiobuccal (MB) canal and mesiolingual (ML) canal mandibular molars and MB1, MB2, D on maxillary molars; S1, S2, F1, F2 to RWL to shape D canal on mandibular molars and P on maxillary molars.
8. Patency phase: Among each rotary instrument, with the pulp-chamber filled with 2.5% NaOCl, a K10, used like patency-file, was taken to RWL + 0.5 mm.
9. Irrigation phase: It was performed using 2.5% NaOCl and pumping technique using GP points in an up-and-down motion at the WL, changing often the irrigant using fresh solution of NaOCl.
10. Intraoperative radiograph with gutta-percha points at WL.
11. Filling phase: It was performed using matched GP points and BioRoot bioceramic sealer.
12. Postoperative radiographs.

### Radiograph Assessment

**Fig. 1:**
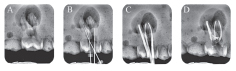
Radiograph assessment for maxillary molar G1. ( **A** ) Preoperative, ( **B** ) intraoperative working length with K file, ( **C** ) intraoperative radiograph with master cones, and ( **D** ) postoperative.

All the radiographs were analyzed independently by two senior endodontists. They assessed radiographically the quality of the root filling and the possible presence of iatrogenic errors. Before the assessment, the two endodontists did not know the protocol used by the anonymous student, with a view to objectivity. When the two examiners were in disagreement, they discussed the case, with a third endodontist, full professor at SU, to solve the problem. The technical quality of root filling and the presence of procedural errors were based on the preoperative WL radiograph determination and postoperative radiographs ( Figs. 1 2 3 4 5 ). The quality of root filling was assessed on two variables: length and density. They were classified as acceptable or unacceptable as follows: 14

**Fig. 2:**
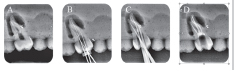
Radiograph assessment for maxillary molar G2. ( **A** ) Preoperative, ( **B** ) intraoperative working length with K file, ( **C** ) intraoperative radiograph with master cones, ( **D** ) postoperative.

**Fig. 3:**
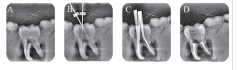
Radiograph assessment for mandibular molar G1. ( **A** ) Preoperative, ( **B** ) intraoperative working length with K file, ( **C** ) intraoperative radiograph with master cones, ( **D** ) postoperative.

**Fig. 4:**
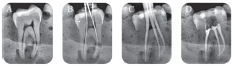
Radiograph assessment for mandibular molar G2. ( **A** ) Preoperative, ( **B** ) intraoperative working length with K file, ( **C** ) intraoperative radiograph with master cones, ( **D** ) postoperative.

**Fig. 5:**
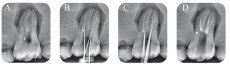
Radiograph assessment for maxillary molar G2 with MB2. ( **A** ) Preoperative, ( **B** ) intraoperative working length with K file, ( **C** ) intraoperative radiograph with master cones, ( **D** ) postoperative.

1. Acceptable: The filling material ends 0 to 2 mm short of the radiographic apex with no voids visible, no overfilling, and with the absence of any procedural error.
2. Unacceptable:
  - Under-filled: The filling material ends more than 2 mm from the radiographic apex.
  - Over-filled: Materials extruded beyond the apex.
  - Voids presence (no adequate density): Voids are visible within or between the material and the root canal walls.

The criteria for radiographic classification of the iatrogenic errors were based on the presence of: 13

1. Ledge: If the filling material was at least 1 mm shorter than the WL and deviated from the original canal shape in teeth where root canal curvature occurred.
2. Apical transportation: If the filling material was located on the outside curve of the canal at the apical third.
3. Perforation: If an extrusion of materials was detected in any area of the root (lateral wall or the foramen of the root).
4. Fractured instrument: If a fractured instrument was detected through observation of the postoperative radiograph.
5. Apical zipping: If the apical termination of the filled canal appeared as an elliptical shape transported to the outer wall.
6. Missed canal: If the filling material was not centered in the root and or there was a radiolucent space indicating presence of another canal. In these cases, access cavity preparation was observed by an operative microscope (Zumax) to confirm or not the presence of an extra canal.

### Statistical Analysis

An analysis per canal and per tooth was performed. When one canal was evaluated below standard, the whole tooth was evaluated below standard. Chi-squared test was performed to investigate the effect of the two different teaching protocols (G1 or G2) used. Data was processed using SigmaPlot (Version 11.2; Systat Software, INC., San Jose, California, United States). In addition, we compared the lapse to complete the RCT (LRCT) and the lapse to perform the root canal filling (LF) of both G1 and G2, using the *t* -test. The first species risk level has been fixed to *p* = 0.05 for all tests performed.

## Results

**Fig. 6:**
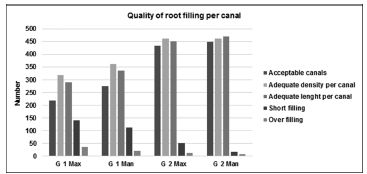
Representative graphic of the quality of root filling per canal treated by G1 and G 2 in maxillary and mandibular molars.

**Fig. 7:**
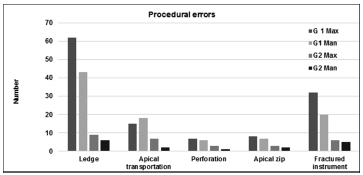
Representative graphic of the procedural errors per canal treated by G1 and G 2 in maxillary and mandibular molars.

At first, each canal was assessed individually to evaluate the quality of root filling and the presence of iatrogenic errors. Four hundred sixty-eight root canals from 152 maxillary molars were treated by the G1 students: 46.6% canals were judged as acceptable, and 53.4% canals were judged as unacceptable. Four hundred sixty-nine root canals from 152 mandibular molars were treated by G1: 58.8% canals were judged as acceptable, and 41.2% canals were judged as unacceptable. Five hundred fifteen root canals from 156 maxillary molars were treated by G2 students: 84.1% canals were judged as acceptable, and 15.9% canals were judged as unacceptable. Four hundred ninety-three root canals from 156 mandibular molars were treated by G2: 90.9% canals were judged as acceptable, and 9.1% canals were judged as unacceptable ( Fig. 6 ). In the present study, the technical quality of root filling per canal and per tooth was significantly higher in G2 than G1 either on maxillary molars or on mandibular molars. For both, G1 and G2, the most common procedural error was ledge, either on maxillary molars or on mandibular molars ( Fig. 7 ).

**Fig. 8:**
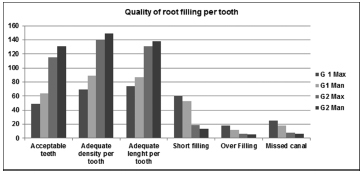
Representative graphic of quality of root filling per tooth treated by G1 and G 2 in maxillary and mandibular molars.

Then the quality of the root filling of each root canal was assessed per tooth; 32.2% maxillary molars and 42.1% mandibular molars were judged as acceptable for G1, whereas 73.7% maxillary molars and 84% mandibular molars were judged as acceptable for G2 ( Fig. 8 ).

Moreover, we evaluated the presence of missed canals per tooth: 27.6% maxillary molars and 21% mandibular molars, for G1, presented a missed canal, whereas 9% maxillary molars and 7% mandibular molars presented a missed canal, for G2. For G1, the LRCT was 127 and 104 minutes, respectively, for maxillary and mandibular molars and LF was 54 and 51 minutes, respectively, for maxillary and mandibular molars. Instead, for G2 the LRCT was 83 and 72 minutes, respectively, for maxillary and mandibular molars, and LF was 28 and 24 minutes, respectively, for maxillary and mandibular molars.

## Discussion

RCT is one of the most common dental interventions, with 7.6 million procedures performed just in France every year.

Considering health and economical repercussions of inadequate RCT, we can conclude that there is an urgent need to find a more suitable procedure to make routine endodontic treatment, on molar tooth, too easier, more reproducible, and manageable even for GPs and for students.

Therefore, it is crucial for each university to be able to give students clinical skills to perform a good standard endodontic therapy on routine multirooted teeth. To face this challenge, it is imperative to recruit endodontic specialists to teach in the preclinical training and in undergraduate clinic. It is essential to use surgical microscopes because of the importance of magnification. 15

It is necessary for the teacher to propose different shaping techniques, to deal with the specificity of each endodontic anatomy This would allow the students to choose the right sequence according to the peculiarity of every single canal.

According to previous studies, anterior or premolar teeth always have a better quality of root filling and a lower rate of procedural errors than molars. 6 16 17 18 That is the reason why we decided to include only the molar teeth in our study. Thanks to the single canal analysis, we were able to evaluate which was the most difficult canal to treat: for G1, the mesiobuccal canal of maxillary and mandibular molars presented a higher rate of procedural errors; for G2, there was no statistical difference in procedural errors. This could mean that the protocol used by G2 would be able to minimize the difference in the treatment of difficult and easy canals. In other words, it could mean that G2 teaching protocol would be able to uniform the canal difficult, transforming a difficult canal to an easier canal.

In the present study, the technical quality of root filling per canal and per tooth was significantly higher in G2 than in G1 either on maxillary molars or on mandibular molars. Furthermore, the frequency of voids presence, overfilling and underfilling per canal in G2 was reduced by 21.3, 5.2, and 20.2% for maxillary molars and 16.7, 3.3, and 20.6% for mandibular molars. First of all, the lower rate of voids presence could be explained by the use of the CHC technique with matched gutta-percha cones and bioceramic sealer. Then the reduced wedging effect for CHC could explain the less presence of overfilling. 19 Finally, the lower rate of procedural errors would explain the lower rate of under-filled canals.

After the technical quality, we assessed the presence of iatrogenic errors.

For G1, the most common procedural errors either on maxillary molars or on mandibular molars were ledges and fractured instruments. For G2, the most common procedural errors on maxillary molars were ledges and apical transportations, whereas on mandibular molars, the most common procedural errors were ledges and fractured instruments.

Among these errors, the incidence of “ledges” and “fractured instruments” was statistically significant in G1, both on maxillary and on mandibular molars, whereas the incidence of “apical transportation” was statistically significant on mandibular molars.

These results might be due to the initial manual scouting in curved canals, especially if it performed by an inexperienced operator, it might more easily cause procedural errors. On the contrary, the step-down technique without initial manual scouting, taught at SU for G2, could eliminate safer and more quickly the coronal and middle interferences of the root canal system, respecting the endodontic anatomy. This technique using at first a rotary NiTi glide path instrument, up to the two-thirds of the root canal length or until to the first impediment, could allow an easy apical scouting of the last millimeters of endodontics. Furthermore, it increases the volume of the irrigants in the apical region, starting from the initial stages of the canal instrumentation, to avoid the most common procedural errors. To perform this mechanical preflaring, Proglider was used instead of a classic opener. The same procedure, using a classic opener with deep, taper and large tip would have been less effective, considering that the opener is made to relocate and negotiate the first 2 or 3 mm of the root canal.

Hence, after the preflaring step, 10 K file worked without any coronal interference in the lasts 2 or 3 mm of the canal, giving a better control during the apical scouting. 20 21

In this way, difficult canals were scouted easily, reaching into the apex and assessing the whole shaping faster and safer.

The second, but not less important, reason was that the new teaching protocol highlighted the importance of preoperative radiographs and preaccess analysis, to plan the appropriate shaping sequence in relation to the root canal anatomy.

So for G2 in case of curve canals, like ML and MB on mandibular molars and MB and D on maxillary molars, the sequence used was S1, S2, F1, PF 25/06. Yet, in case of very curve canals, the sequence used was S1, S2, followed by profile 25/04, whereas for G1 the sequence used was always the same, S1, S2, F1, F2. Moreover, G2 was taught to detect the merged canals, like MB and ML, on mandibular molars or MB1 and MB2 on maxillary molars. For merged canals, after the glide path, the easier canal was shaped until WL, the other one until the merged point. This allowed to avoid apical zips or hazardous stress for the endodontic instrument.

The rate of missed canals was statistically higher for G1, in both mandibular and maxillary molars. This finding could be explained by the fact that in the new teaching protocol of G2, after the access cavity opening, the access cavity preparation was checked with a clinical operative microscope. 15

Finally, the LRCT and the LF were significantly shorter for G2 ( *p* < 0.001) in both mandibular and maxillary molars, confirming that the new teaching protocol could facilitate the endodontic treatment of molar teeth and make them more reproducible and manageable even for a student.

Even though the results of this study are quite interesting, we have to consider that it is only an observational study on preclinical training. The higher success rate achieved for G2 could confirm that the new endodontic teaching is superior to the standard protocol. The former uses the surgical microscope to check the access cavity, a step-down technique without initial manual scouting for the shaping, with a different protocol according to endodontic anatomy, and a CHC technique for the obturation. This study could prove that an endodontic treatment for molars can be reproducible even if performed by an inexperienced operator provided that he uses a strict protocol respecting the basic principles to perform correctly an endodontic therapy. In other words, the educational changes resorted at SU Department of Endodontics allowed to improve, beyond all doubt, the quality of root canal filling. However, to show definitively if the new teaching protocol facilitates the endodontic treatment with a concomitant improvement in periodontal status, in the near future, we will compare the two teaching protocols in vivo, too, in Strasbourg Dental Teaching Hospital, during the students’ clinical training on patients.
